# Expression and Characterization of a β-Galactosidase from the Pacific Oyster, *Crassostrea gigas,* and Evaluation of Strategies for Testing Substrate Specificity

**DOI:** 10.3390/ijms242015287

**Published:** 2023-10-18

**Authors:** Julia Thoma, Reingard Grabherr, Erika Staudacher

**Affiliations:** 1 Department of Chemistry (DCH), University of Natural Resources and Life Sciences, 1190 Vienna, Austria; julia.thoma@boku.ac.at; 2 Department of Biotechnology (DBT), University of Natural Resources and Life Sciences, 1190 Vienna, Austria; reingard.grabherr@boku.ac.at

**Keywords:** β-galactosidase, exoglycosidase, *Crassostrea gigas*, *Arion vulgaris*, Mollusca, High-Performance Thin-Layer Chromatography (HPTLC), enzyme activity determination

## Abstract

β-Galactosidases (EC 3.2.1.23) are exoglycosidases that catalyze the cleavage of glycoconjugates with terminal β-D-galactose residues in β1,3-, β1,4- or β1,6-linkage. Although this family of exoglycosidases has been extensively studied in vertebrates, plants, yeast, and bacteria, little information is available for mollusks. Mollusks are a diverse and highly successful group of animals that play many different roles in their ecosystems, including filter feeders and detritivores. Here, the first β-galactosidase from the Pacific oyster, *Crassostrea gigas* was discovered, biochemically characterized, and compared to our previously characterized slug enzyme from *Arion vulgaris* (UniProt Ref. Nr.: A0A0B7AQJ9). Overall, the mussel enzyme showed similar biochemical parameters to the snail enzyme. The enzyme from *C. gigas* was most active in an acidic environment (pH 3.5) and at a reaction temperature of 50 °C. Optimal storage conditions were up to 37 °C. In contrast to the enzyme from *A. vulgaris*, the supplementation of cations (Ni^2+^, Co^2+^, Mn^2+^, Mg^2+^, Ca^2+^, Cu^2+^, Ba^2+^) increased the activity of the enzyme from *C. gigas*. Substrate specificity studies of the β-galactosidases from the mussel, *C. gigas,* and the slug, *A. vulgaris*, revealed activity towards terminal β1,3- and β1,4-linked galactose residues for both enzymes. Using the same substrates in labeled and unlabeled form, we were able to detect the effect of labeling on the β-galactosidase activity using MALDI-TOF MS, HPTLC, and HPLC. While lactose was cleaved by the enzymes in an unlabeled or labeled state, galacto-N-biose was not cleaved as soon as a 2-amino benzoic acid label was added. In this study we present the biochemical characterization of the first recombinantly expressed β-galactosidase from the Pacific oyster, *C. gigas*, and we compare different analytical methods for the determination of β-galactosidase activity using the enzyme from *C. gigas* and *A. vulgaris*.

## 1. Introduction

Protein glycosylation is a fundamental post-translational modification that is essential for various cellular functions such as cell adhesion, signaling, and immune responses. Various enzymes are responsible for proper biosynthesis and catabolism of specific glycans [1]. One important enzyme is the β-galactosidase (EC 3.2.1.23), which plays a crucial role in the hydrolysis of glycans containing terminal non-reducing β-D-galactose residues in β1,3-, β1,4- or β1,6-linkage [2]. In humans, deficiencies in β-galactosidase activity have been linked to the autosomal recessive disorders GM1 gangliosidosis and Morquio B syndrome [3]. 

While glycosidases and glycosyltransferases have been extensively studied in vertebrates, plants, yeast, and bacteria, our understanding of the glycosylation machinery in mollusks is still rudimentary. This is particularly surprising when looking at the remarkable evolutionary success of mollusks, being the second-largest phylum of invertebrates. Mollusks have very heterogeneous morphologies (gastropods, cephalopods, bivalves) and play many different roles in their ecosystems, including filter feeders and detritivores. However, some species also occur as agricultural pests or as intermediate hosts for human parasites [4].

*Arion vulgaris*, also known as the Spanish slug, is a land snail found in temperate and humid climates of Europe and North America. As they feed on vegetables, field crops, and fruit trees, they represent one of the most pernicious slugs. Their high reproductive potential makes it especially difficult to control their population. Research on these snails is therefore mainly concerned with their reduction in agriculture by means of chemical or biological methods [5]. 

*Crassostrea gigas* also known as the Pacific oyster is the most widely consumed oyster in the world. It is native to the Pacific coast of Asia. To meet the growing demand of the food market, it has been cultivated on an industrial scale, mainly in Korea, Japan, and China. *C. gigas* is especially valued due to its nutritional and bioactive properties. Oysters are an excellent source of protein, polysaccharides, essential amino acids, fatty acids, and minerals. However, they are also known for their antimicrobial, antioxidant, antitumor, or anti-inflammatory activity [6]. So far, little information is available about the glycosylation machinery in oysters, although, unlike other mollusks (*A. vulgaris*) this species is suitable as a model organism since parts of its genome have already been sequenced [7]. In addition, the related species *C. virginica* often serves as an intermediate host for the protozoan *Perkinsus marinus* [8]. In general, investigations on mollusks and their glycosylation capabilities might contribute to a better understanding of their role as agricultural pests or intermediate hosts for parasites.

Similar to vertebrates, mollusks have a broad spectrum of glycosylation capabilities, which is reflected by their heterogenous N- and O-glycan patterns [9,10]. However, until now only a few glycoenzymes have been identified and characterized in mollusks. β-Galactosidases have been extensively studied in vertebrates, plants, and microorganisms. They exhibit diverse enzyme properties such as pH, temperature, or substrate specificity and are therefore used on an industrial scale for the modification of oligosaccharides in the food industry [11,12]. These enzymes are commonly used to improve the sweetness, solubility, taste, or digestibility of dairy products. In particular, the removal of lactose by β-galactosidases is used in dairy products to make them edible for people with lactose intolerance. In addition to the food industry, β-galactosidases are used in a variety of other applications, such as pharmaceutical production and biotechnology laboratories [11]. The most widely studied β-galactosidase is undoubtedly that from *E. coli*, which is often used in molecular biology as a reporter marker to assess gene expression by a technique known as α-complementation [13,14]. This assay is based on the chromogenic substrate X-gal, which is hydrolyzed to 5-bromo-4-chloroindoxyl and galactose by the β-galactosidase. When the 5-bromo-4-chloroindoxyl dimerizes, it produces an insoluble blue pigment called 5,5′-dibromo-4,4′-dichloroindigo [15]. In addition to colorimetric detection methods, active β-galactosidases can be detected in vivo by fluorescence, bioluminescence, chemiluminescence, magnetic resonance (MR), single photoemission computed tomography or positron emission tomography (PET) [16]. Similar to in vivo detection, in vitro determination of β-galactosidase activity is often performed using chromogenic or fluorogenic labeled substrates [17,18,19]. The products are usually detected by a photometer or fluorimeter, respectively. Since the use of substrate labels carries the risk of enzyme inhibition, glycosidase activity can also be measured using unlabeled substrates. The analysis of the unlabeled products is typically carried out by MALDI-TOF MS [20], HPLC equipped with a differential refractometer, or through enzymatic assays that react with the product [21]. While MALDI-TOF MS is a highly sensitive and specific method, the analysis of small molecules (like lactose) can be affected by the presence of low-mass matrix-related ions [22]. However, HPLC equipped with a differential refractometer has its drawbacks, especially when high sensitivity and selectivity are required. Another often underestimated method for the detection of glycosidase activity on unlabeled substrates is the separation of the product by Thin-Layer Chromatography (TLC) or High-Performance Thin-Layer Chromatography (HPTLC) [23]. For the general detection of β-galactosidase activity, HPTLC offers a sensitive, affordable, and, above all, rapid alternative to MALDI-TOF MS, HPLC, or enzyme-based assays.

In this study, we present the identification, expression, and characterization of the first β-galactosidase from the Pacific oyster, *Crassostrea gigas*. The biochemical characterization was based on the chromogenic substrate pNP-β-Gal and the results were compared to our previously characterized β-galactosidase from the slug *Arion vulgaris* [19]. Furthermore, we determined the substrate specificity of the β-galactosidases from *C. gigas* and *A. vulgaris* using MALDI-TOF MS, an enzyme-based assay (Galactose Assay Kit–ab83382; ABCAM, Cambridge, UK), HPTLC and HPLC. Thereby, we obtained an overview of the influence of labels on the enzymes’ activity.

## 2. Results and Discussion

### 2.1. Identification and Expression of Putative β-Galactosidases from C. gigas

**Figure 1:**
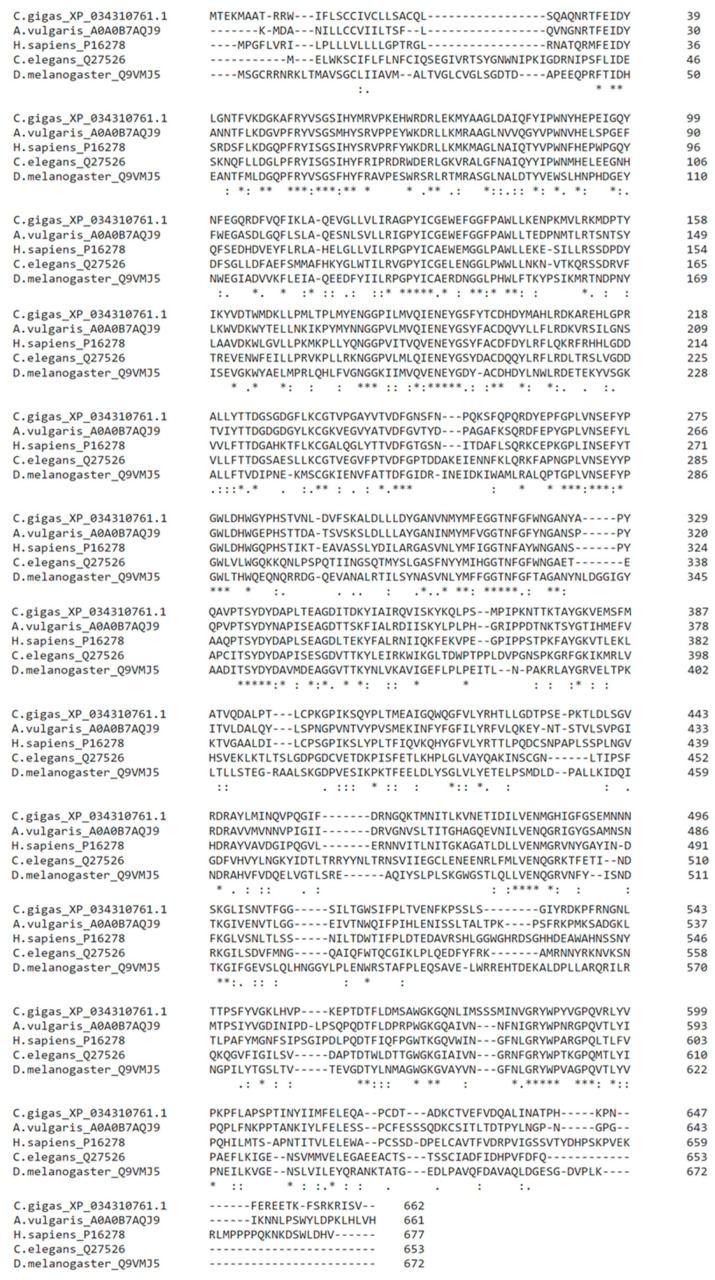
Comparison of β-galactosidase protein sequences from different species (*C. gigas*: XP_034310761.1, *A. vulgaris*: A0A0B7AQJ9, *H. sapiens*: P16278, *C. elegans*: Q27526, *D. melanogaster*: Q9VMJ5). Asterisks (*) indicate positions that have a single, fully conserved residue; colons (:) indicate conservation between groups of strongly similar properties, and periods (.) indicate conservation between groups of weakly similar properties.

Within our previous paper [19] we were able to identify, express, and characterize the first β-galactosidase from mollusk origin. Using this enzyme from the Spanish slug, *Arion vulgaris* (UniProt Ref. Nr.: A0A0B7AQJ9) as the template for homology searches, we were able to identify putative β-galactosidase gene sequences from the Pacific oyster, *Crassostrea gigas*. Overall, we identified 4 isoforms, of which isoform X4 (662 AA, NCBI Ref. Nr.: XP_034310761.1) had the highest sequence similarity to the enzyme from *A. vulgaris* (54.22% identity) and showed further sequence homologies to previously identified β-galactosidases (*H. sapiens*–50.78%, *C. elegans*–37.24%, *D. melanogaster*–38.49%; Figure 1).

The putative full-length β-galactosidase sequence from *C. gigas* (NCBI Ref. Nr.: XP_034310761.1) was selected and synthesized for the expression in Sf9 insect cells using the baculoviral expression system. The recombinant construct encoded 691 amino acids, including a hexa-His-tag, resulting in a protein with a molecular weight of approximately 78 kDa and a calculated isoelectric point (pI) at 6.45.

The presence and activity of the expressed β-galactosidase protein was detected in supernatant (secreted proteins) as well as in lysate fractions (soluble, non-secreted proteins) by Western Blot analysis and activity assay. The purification of the secreted protein (supernatant) on a HisTrap^TM^ excel column was not successful, as the protein lost activity after elution with imidazole. The attempt to elute the enzyme at an acidic pH also failed, as the enzyme was not released from the column. Instead, we performed immunoprecipitation of the lysate fraction, using mouse anti-Penta-Histidine Tag:HRP monoclonal antibodies and elution at acidic pH (Figure S1).

### 2.2. Biochemical Parameters of the Recombinant β-Galactosidase from C. gigas

**Figure 2:**
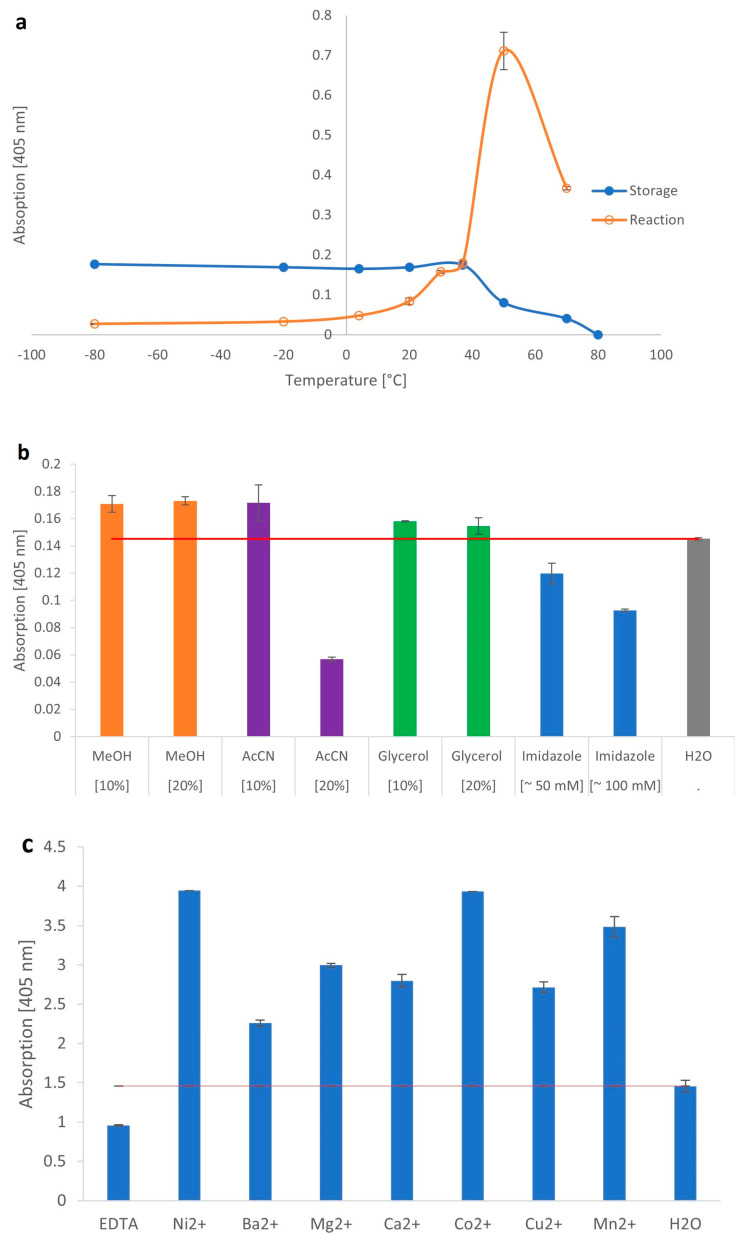

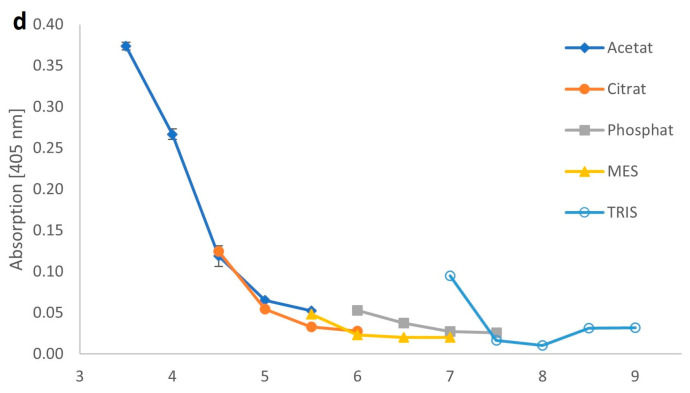
Biochemical properties of the β-galactosidase from *C. gigas*. (**a**) Storage (blue) and reaction (orange) temperature. (**b**) Storage in the presence of methanol (MeOH), acetonitrile (AcCN), glycerol, imidazole and H_2_O. The red line represents control without the addition of chemicals. (**c**) Cation requirement. The red line represents control without the addition of cations (H_2_O). (**d**) Optimal pH environment using different buffer salts. Data points represent mean values of duplicates with corresponding standard deviations.

The biochemical parameters of the β-galactosidase from *C. gigas* were determined using the artificial substrate, pNP-β-Gal. The optimal storage temperature of the recombinant β-galactosidase was in a range from −80 to 37 °C, as temperatures above 37 °C drastically reduced the activity. The optimal reaction temperature for short assays up to 2 h was at 50 °C (Figure 2a). The temperature optimum was similar to other mollusk species, as shown for the slug *Arion vulgaris* (50 °C) [19] or the land snail *Achatina achatina* (50 °C) [24]. For fungal enzymes, an optimal reaction temperature of 4–67 °C has been determined, while most bacterial enzymes prefer temperatures between 40–65 °C [11,12,25]. Overall, the temperature optimum for β-galactosidases varies greatly between species. To investigate the storage stability in different solvents, the recombinant enzyme was stored in methanol, acetonitrile, glycerol, or imidazole. Similar to the β-galactosidase from *A. vulgaris* [19], the activity was mostly affected by 20% (*v*/*v*) acetonitrile, as the enzyme was drastically reduced in activity, to approximately 40%. Furthermore, we identified a reduction of activity by imidazole [100 mM] during short-term incubation (2 h, 37 °C). The sensitivity to imidazole is particularly noteworthy, as it explained the problems encountered with purification using the HisTrap^TM^ excel column. However, the enzyme was fully active in the presence of methanol or glycerol (Figure 2b). 

Regarding the requirement for divalent cations, the β-galactosidase of *C. gigas* was significantly improved with the order of decreasing effect being Ni^2+^ > Co^2+^ > Mn^2+^ > Mg^2+^ >Ca^2+^ > Cu^2+^ > Ba^2+^. However, the enzyme did not necessarily require cations for its activity, as the presence of EDTA only reduced, but did not completely inhibit the enzyme (Figure 2c). In contrast, for the enzyme from *A. vulgaris,* we were able to show the independence of cations [19]. While in the microorganism *Bacillus stearothermophilus,* most cations had no effect on the activity of the enzyme, in *E. coli* the enzyme showed a clear requirement for Mg^2+^ and Mn^2+^ [26,27]. Generally, it is known from many species that the cation requirement of β-galactosidases is very heterogeneous. 

The optimal pH for the β-galactosidase from *C. gigas* was at pH 3.5 using acetate as the buffer salt (Figure 2d), which correlates with other mollusk β-galactosidases that range between pH 3.2 and 5.6 [19,24,28,29,30].

During inhibition studies, the activity of the recombinant protein was tested in the presence of different monosaccharides (GlcNAc, GalNAc, Gal, Glc). Thereby, minor product inhibition was detected by the addition of 6 mM and 12 mM galactose, which resulted in an approximately 20% and 25% reduction of activity, respectively. All other monosaccharides did not show any inhibitory effects. Galactose inhibition was also detected for our previously characterized β-galactosidase from *A. vulgaris* [19].

### 2.3. Substrate Specificity of the Recombinant β-Galactosidase from C. gigas

Four methods were used to identify the linkage specificity of the β-galactosidase towards unlabeled and labeled substrates: (i) MALDI-TOF MS for large substrates, (ii) an enzyme-based Galactose Assay Kit (ab83382; ABCAM–Cambridge, UK) for unlabeled substrates, (iii) HPTLC for small unlabeled substrates and (iv) HPLC for small, labeled substrates.

Depending on the length of the sugar chains, the substrates were either labeled with 2-aminopyridine (PA; oligosaccharides) or 2-aminobenzoic acid (AA; mono/disaccharides) and analyzed by MALDI-TOF MS or HPLC, respectively. The corresponding unlabeled substrates were analyzed by MALDI-TOF MS, an enzyme-based Galactose Assay Kit or HPTLC.

#### 2.3.1. MALDI-TOF MS Analysis of Labeled and Unlabeled Substrates

**Figure 3:**
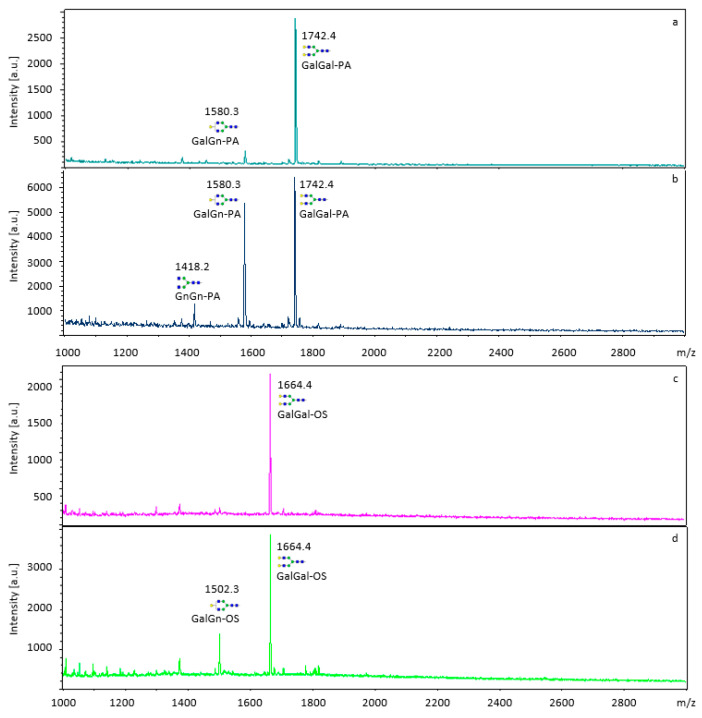
MALDI-TOF analysis of β-galactosidase activity towards the N-glycan, GalGal-OS, and GalGal-PA. (**a**) GalGal-PA substrate, (**b**) GalGal-PA incubated with purified β-galactosidase from *C. gigas*, (**c**) GalGal-OS substrate, (**d**) GalGal-OS incubated with purified β-galactosidase from *C. gigas*. N-Glycan structures were created using bioRENDER.

The β-galactosidase activity from *C. gigas* was tested on the natural, unlabeled substrates lacto-N-fucopentaose (Lewis-A antigen) and the N-glycan GalGal-OS. The same substrates were also labeled with 2-aminopyridine (lacto-N-fucopentaose II-PA and GalGal-PA). In addition, β-galactosidase activity was tested towards the 2-aminopyridine labeled oligosaccharides derived from galactan: Gal_4_-PA and Gal_8_-PA. All substrates were incubated with the oyster β-galactosidase and the release of galactose was detected by analyzing the product on MALDI-TOF MS. Analysis of the MALDI-TOF MS spectra indicated, that the β-galactosidase was active on both, the unlabeled and labeled forms of the N-glycan, GalGal-OS, and GalGal-PA, respectively (Figure 3). The enzyme did not show activity neither towards the galactan-derived Gal_4_-PA and Gal_8_-PA sugar chains nor the unlabeled nor labeled lacto-N-fucopentaose II (lacto-N-fucopentaose II-OS, lacto-N-fucopentaose II-PA), as for both sugars the terminal galactose was not released from the substrate (Figures S2 and S3).

The attachment of a 2-aminopyridine (PA) label to these substrates had no effect on the enzyme’s activity, probably because the label was distant from the active site of the enzyme.

Overall, MALDI-TOF MS is a highly sensitive and specific method suitable for the detection of unlabeled or labeled substrates. However, limitations occur when analyzing small sugars (mono- and disaccharides) due to the presence of matrix-related ions in the low-mass region of the spectrum [22].

#### 2.3.2. Unlabeled Substrates Tested Using the Galactose Assay Kit (ab83382; ABCAM)

To determine β-galactosidase specificity for small unlabeled sugar chains, we used the enzyme-based Galactose Assay Kit from ABCAM (ab83382). According to the product datasheet, released galactose gets enzymatically oxidized forming a product that reacts with the galactose probe to produce color (OD570 nm) or fluorescence (Ex/Em 535/587 nm). Thereby, Gal levels can be directly measured from various biological samples such as serum or growth media without prior purification. Analyzing the monosaccharide Gal at different concentrations, we obtained a linear calibration curve (Figure S4c). While the monosaccharides Glc, GlcNAc, Fuc, and Man did not show any staining using the kit, the monosaccharide GalNAc was clearly detectable (Figure S4a). Furthermore, the putative substrates for the β-galactosidase, the disaccharides lactose, lacto-N-biose, galacto-N-biose, Galβ1,6GlcNAc and N-acetyllactosamine, were detected by the kit (Figure S4b), even when Gal was linked to other monosaccharides. Therefore, we have to emphasize that the kit is not suitable for measuring free galactose levels, after β-galactosidase activity, because not only the product but also the substrates are detected.

#### 2.3.3. Unlabeled Substrates Tested Using High-Performance Thin-Layer Chromatography (HPTLC)

HPTLC is a suitable method for the determination of β-galactosidase activity for small, unlabeled substrates such as lactose, galacto-N-biose, N-acetyllactosamine, Galβ1,6GlcNAc or 3-fucosyllactose and was thus, included in our study.

**Figure 4:**
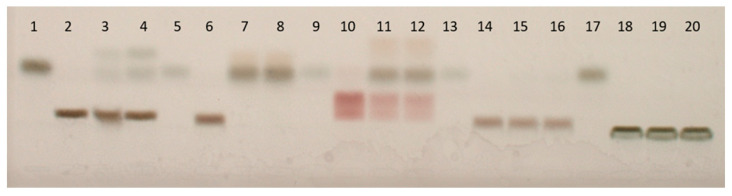
Substrates incubated with the β-galactosidases from *C. gigas* or *A. vulgaris* (UniProt Ref. Nr.: A0A0B7AQJ9) [19] after development and visualization at white light. (1) galactose 1 μg/μL, (2) lactose (control), (3) lactose + enzyme (*A. vulgaris*), (4) lactose + enzyme (*C. gigas*), (5) galactose 0.25 μg/μL, (6) galacto-N-biose (control), (7) galacto-N-biose + enzyme (*A. vulgaris*), (8) galacto-N-biose + enzyme (*C. gigas*), (9) galactose 0.25 μg/μL, (10) N-acetyllactosamine (control), (11) N-acetyllactosamine + enzyme (*A. vulgaris*), (12) N-acetyllactosamine + enzyme (*C. gigas*), (13) galactose 0.10 μg/μL, (14) Galβ1,6GlcNAc (control), (15) Galβ1,6GlcNAc + enzyme (*A. vulgaris*), (16) Galβ1,6GlcNAc + enzyme (*C. gigas*), (17) galactose 0.50 μg/μL, (18) 3-fucosyllactose (control), (19) 3-fucosyllactose + enzyme (*A. vulgaris*), (20) 3-fucosyllactose + enzyme (*C. gigas*).

All substrates were incubated with the β-galactosidase from *C. gigas* or *A. vulgaris*. The reaction mixtures were spotted on the HPTLC plate and compared with unprocessed substrate and galactose standards. Through the application of unprocessed substrates, the background for each substrate was determined. By using galactose standards, the correct retention of galactose could be determined in the case of degradation by β-galactosidase. Both β-galactosidases, the enzyme from *C. gigas* as well as from *A. vulgaris*, degraded galacto-N-biose completely (Figure 4, lane 7–9), while lactose and N-acetyllactosamine were partly degraded (Figure 4, lanes 2–4 and 10–12 respectively). Again, both enzymes showed identical patterns. No degradation was detected for Galβ1,6GlcNAc and 3-fucosyllactose by any of the two mollusk enzymes (Figure 4, lanes 14–16 and 18–20 respectively).

By using unlabeled substrates, we were able to demonstrate β-galactosidase activity towards terminal β1,3- and β1,4-linked galactose residues by the enzymes of the Pacific oyster *C. gigas* and the snail *A. vulgaris*.

#### 2.3.4. Labeled Substrates Tested Using High-Performance Liquid Chromatography (HPLC)

In addition to the activity of the β-galactosidase towards different substrates with terminal galactose residues, the specificity towards different monosaccharides was also tested. Thereby, the following artificial pNP-subtrates were used: pNP-β-Gal, pNP-α-Gal, pNP-α-Glc, pNP-β-Glc, pNP-α-GalNAc, pNP-β-GalNAc, pNP-β-GlcNAc, pNP-α-Fuc, pNP-α-Man, pNP-β-Man. 

Results showed that the enzyme cleaved pNP-β-Gal very well and pNP-α-Glc to some extent. All other monosaccharides were not affected.

Furthermore, we compared the β-galactosidase substrate specificity of unlabeled substrates (determined by HPTLC; Figure 4) with the same substrates labeled with 2-amino benzoic acid or pNP (determined by HPLC). Interestingly, of the AA-labeled substrates (lactose-AA, galacto-N-biose-AA, N-acetyllactosamine-AA, Galβ1,6GlcNAc-AA, 3-fucosyllactose-AA), only lactose-AA was susceptible to enzymatic cleavage. All other AA-labeled substrates were not cleaved by the enzyme. In addition, cleavage was also observed for the pNP-substrates, pNP-lactose, and pNP-galacto-N-biose (Table 1). The other substrates were not available with a pNP-label. Identical results were obtained for the β-galactosidase from *A. vulgaris*.

Based on the results generated by HPTLC and HPLC, it can be concluded that the type of label (AA, pNP) has a serious impact on the accessibility of the substrate to the enzyme. The exact conformation mechanism of the inhibition by the label on the activity of the β-galactosidases still needs to be investigated in more detail. Overall, the determination of substrate specificity of an enzyme using labeled substrates must be interpreted with caution if the enzyme shows no activity. Therefore, a combination of test strategies is necessary. Especially sensitive detection techniques that allow the use of unlabeled substrates are indispensable.

## 3. Materials and Methods

### 3.1. Materials

Q5/Taq DNA Polymerases, restriction enzymes, and T4 ligase were purchased from New England Biolabs (Frankfurt, Germany). All enzymes were used according to the supplier’s instructions. Primers and gBlock gene fragments were synthesized commercially at Sigma-Aldrich (Vienna, Austria) and Integrated DNA Technologies (Leuven, Belgium), respectively. pACEBac1 vector was purchased from Geneva Biotech (Genève, Switzerland).

All other chemicals and molecular biology reagents were of the highest quality available and purchased from Sigma-Aldrich (Vienna, Austria), Merck (Darmstadt, Germany), Roth (Karlsruhe, Germany), Honeywell (Vienna, Austria) and ThermoFisher Scientific (Bonn, Germany) unless indicated otherwise.

Electrocompetent *E. coli* cells–Neb5α (NEB Frankfurt, Germany) were spread on Lysogeny Broth (LB) agar plates containing 15 µg/mL gentamycin and incubated overnight at 37 °C. Electrocompetent *E. coli* cells–DH10EMBacY cells (Geneva Biotech–Genève, Switzerland) were cultivated on Lysogeny Broth (LB) agar plates containing 15 µg/mL gentamycin, 50 µg/mL kanamycin, 10 µg/mL tetracycline, 50 µg/mL IPTG, 100 µg/mL X-gal and incubated for 2 days at 37 °C. Electroporation was done using a MicroPulser from BIORAD.

*Spodoptera frugiperda* cells–Sf9 (ATCC Manassas Virginia, United States) were grown in SFM4Insect media with L-Glutamine (HyClone Cytiva–Vienna, Austria) and kept at 27 °C [31]. Viable cell numbers were determined using the Vi-CellTM XR cell viability analyzer (Beckman Coulter–Vienna, Austria).

Substrates for β-galactosidases, galactan, and lacto-N-fucopentaose II, were bought from Megazyme (Wicklow, Ireland) and Oxford GlycoSystems (Bedford, UK) respectively, and labeled with 2-aminopyridine (PA) according to [32]. pNP-sugars were obtained from Sigma-Aldrich (Vienna, Austria), mono- and disaccharides were from Sigma-Aldrich and labeled with 2-amino benzoic acid (AA) according to [33].

### 3.2. Expression of the Full-Length β-Galactosidase Gene from C. gigas

We identified four β-galactosidase isoforms X1-X4 within the *C. gigas* genome trough BLASTp search (NCBI) using the Mollusca database (taxid: 6447) and the β-galactosidase from *A. vulgaris* (UniProt Ref. Nr.: A0A0B7AQJ9) as a template [19]. The protein with the highest similarity score to *A. vulgaris* (NCBI Ref. Nr.: XP_034310761.1) was chosen and modified with a C-terminal hexahistidine-tag and the N-terminal gp64 secretion signal sequence MVSAIVLYVLLAAAAHSAFA (Figure S5) [34].

Modification and amplification of the gene was done using PCR, using the forward primer 5′GATGATGAATTCATGGTGTCTGCTATTGTTCTG3′ and the reverse primer 5′GATGATTCTAGATTAATGATGGTGGTGATGATGG3′.

Protein expression in Sf9 insect cells was performed according to [19]. Purification was done through immunoprecipitation using mouse anti-Penta-Histidine Tag:HRP monoclonal antibodies (BIORAD–Vienna, Austria) linked to protein A/G-plus agarose beads (CALBIOCHEM–San Diego, United States). Elution of the recombinant protein from the beads was achieved at an acidic pH (0.2 M acetate buffer, pH 3.5). The purified β-galactosidase was analyzed by SDS-PAGE and Western blot using the mouse anti-Penta-Histidine tag:HRP monoclonal antibody (1:2500, BIORAD–Vienna, Austria) followed by alkaline phosphatase-conjugated anti-mouse IgG from goat (1:4000, Sigma-Aldrich, Vienna, Austria) [19]. 

### 3.3. Determination of β-Galactosidase Activity from C. gigas

The analysis of β-galactosidase activity was based on a colorimetric assay using the artificial substrate 4-Nitrophenyl β-D-galactopyranosid (pNP-β-Gal, Merck Darmstadt, Germany). The reaction was performed in 50 µL containing 5 µL of enzyme solution (~0.5 μg enzyme in 0.2 M acetate buffer, pH 3.5), 20 µL of 0.9% NaCl-solution and 25 µL of pNP-β-Gal substrate (5 mM pNP-β-Gal in 0.1 M NaCitrat buffer, pH 4.5) at 37 °C for 2 h. The reaction was terminated by adding 200 µL of glycine/NaOH (0.4 M, pH 10.4). The absorbance of the released *p*-nitrophenol was measured at 405 nm. For analysis of the biochemical parameters, the standard assay conditions using pNP-β-Gal as the substrate were modified as follows. For the determination of cation requirement, the standard assay was carried out without any cation addition or in the presence of 20 mM of EDTA, Mn^2+^, Mg^2+^, Ca^2+^, Co^2+^, Cu^2+^, Ni^2+^, or Ba^2+^. Corresponding cation blanks were performed for Ni^2+^, Co^2+^ and Cu^2+^ to avoid bias from the colored metal solutions. The chemical stability of the enzyme, optimal storage conditions, and pH-optimum were processed according to [19]. For storage stability in chemicals, the enzyme was incubated for approximately 16 h in 10% or 20% of methanol, acetonitrile, glycerol, or imidazole [50 mM or 100 mM]. For inhibition studies the standard assay was performed in the presence of 6 mM or 12 mM monosaccharide (GlcNAc, GalNAc, Gal, Glc,). The substrate specificity of the β-galactosidase towards different artificial pNP-monosaccharides (pNP-α-Gal, pNP-α-Glc, pNP-β-Glc, pNP-α-GalNAc, pNP-β-GalNAc, pNP-β-GlcNAc, pNP-α-Fuc, pNP-α-Man, pNP-β-Man) was tested under the same standard conditions as described above. Each assay was performed at least in duplicate with appropriate controls.

### 3.4. Galactan Hydrolysis

2 mg of galactan ([-Galβ1,4Galβ1,4-]_n_) was dissolved in 400 μL of 0.5 M HCl and incubated at 100 °C for 2 h before immediate neutralization with NaOH. The obtained Gal_4_–Gal_8_-chains were labeled with 2-aminopyridine (PA), before fractionation by size on HPLC.

### 3.5. Substrate Specificity Assay

Substrate specificity of β-galactosidases (*C. gigas* and *A. vulgaris* [19]) was performed with 1–5 μg dry substrates (unlabeled or labeled) and 5 µL enzyme (~0.5 μg of β-galactosidase in 0.2 M acetate buffer, pH 3.5) at 37 °C over-night.

#### 3.5.1. MALDI-TOF MS Analysis

MALDI-TOF MS analysis of unlabeled/labeled substrates (GalGal-OS, GalGal-PA, Lacto-N-fucopentaose II(-PA) and Gal_4_-PA/Gal_8_-PA chains derived from galactan) were performed on an Autoflex Speed MALDI-TOF (Bruker Daltonics Bremen, Germany) equipped with a 1000 Hz Smartbeam.II laser in positive mode using 2% (*w*/*v*) dihydroxybenzoic acid in 50% (*v*/*v*) acetonitrile as the matrix. Spectra were processed with the manufacturer’s software (Bruker Flexanalysis 3.3.80).

#### 3.5.2. Enzyme-Based Galactose Assay Kit (ab83382; ABCAM–Cambridge, UK)

For analysis of unlabeled substrates (lactose: Galβ1,4Glc; galacto-N-biose: Galβ1,3GalNAc; 2-fucosyllactose: Fucα1,2Galβ1,4Glc; 3-fucosyllactose: Fucα1,3[Galβ1,4]Glc; N-acetyllactosamine: Galβ1,4GlcNAc and Galβ1,6GlcNAc), approximately 5 µg of dry substrate were incubated with 5 µL of enzyme (~0.5 μg of β-galactosidase in 0.2 M acetate buffer, pH 3.5) at 37 °C over-night. The galactose release was tested using a commercially available Galactose Assay Kit (ab83382; ABCAM–Cambridge, UK) according to the supplier’s instructions. Each assay was performed in duplicates with the appropriate controls (assay without enzyme).

#### 3.5.3. High-Performance Thin-Layer Chromatography (HPTLC) Analysis

The specificity of β-galactosidases towards unlabeled substrates (lactose: Galβ1,4Glc; galacto-N-biose: Galβ1,3GalNAc; 2-fucosyllactose: Fucα1,2Galβ1,4Glc; 3-fucosyllactose: Fucα1,3[Galβ1,4]Glc; N-acetyllactosamine: Galβ1,4GlcNAc and Galβ1,6GlcNAc) was examined on a HPTLC plate silica gel 60 F254 (MERCK Darmstadt, Germany) by applying 2 μL of the reaction assay or galactose standards (0.1–1 µg/µL) via a Linomat IV (CAMAG Muttenz, Switzerland). The reaction assay consisted of 1 μg dry substrate incubated with ~0.5 μg of β-galactosidase in 0.2 M acetate buffer, pH 3.5 at 37 °C, over-night. The following settings have been selected for 20 tracks per plate: band length 6.0 mm, track distance 2.5 mm, dosage speed 4 sec/μL, application position x-axis 13.0 mm and y-axis 0.8 mm, application volumes 2 μL. The plate was run in a horizontal developing chamber (CAMAG Muttenz, Switzerland) using n-butanol-i-propanol-acetic acid-boric acid solution (200 mg boric acid dissolved in 10 mL entionized water) 6/14/1/3 (*v*/*v*/*v*) as the mobile phase, up to a migration distance of 60 mm. After a dry time of 15 min, the plate was immersed manually into aniline diphenylamine o-phosphoric acid reagent (mixture of 70 mL aniline solution, 70 mL diphenylamine solution, both 2% each in acetone, and 10 mL *o*-phosphoric acid, 85%). Subsequently, the HPTLC plate was heated on a glass-ceramic hot plate at 120 °C for 5 min.

Plate images were documented by TLC Visualizer (CAMAG Muttenz, Switzerland) using white light. CAMAG instruments were controlled with VisionCats v1.4. (CAMAG Muttenz, Switzerland) [35].

#### 3.5.4. High-Performance Liquid Chromatography (HPLC) Analysis

The substrate specificity of the mussel (*C. gigas*) and snail (*A. vulgaris*) β-galactosidases towards 2-amino benzoic acid labeled di- and trisaccharides (lactose-AA: Galβ1,4Glc-AA; galacto-N-biose-AA: Galβ1,3GalNAc-AA; 2-fucosyllactose-AA: Fucα1,2Galβ1,4Glc-AA; 3-fucosyllactose-AA: Fucα1,3[Galβ1,4]Glc-AA; N-acetyllactosamine-AA: Galβ1,4GlcNAc-AA and Galβ1,6GlcNAc-AA) were analyzed on reverse-phase HPLC (ODS HypersilTM, 250 × 4 mm, ThermoFisher Scientific–Bonn, Germany) with solvent A: 0.2% (*v*/*v*) 1-butylamin, 0.5% (*v*/*v*) orthophosphoric acid, 1% (*v*/*v*) tetrahydrofuran in H_2_O and solvent B: solvent A/acetonitrile = 50/50 (*v*/*v*). The elution was performed by a linear gradient of solvent B from 5–100% in 23 min, at a flow rate of 1 mL/min. Quantification was done by peak integration after fluorescence detection at ex/em 360 nm/425 nm [33].

Separation of pNP-labeled sugars (pNP-lactose: pNP-Glcβ1,4Gal, pNP-galacto-N-biose: pNP-GalNAcβ1,3Gal) was done on reverse-phase HPLC (ODS HypersilTM, 250 × 4.6 mm, ThermoFisher Scientific–Bonn, Germany) with solvent A composing of 0.1 M ammonium acetate, pH 6.0 and solvent B containing 50% (*v*/*v*) acetonitrile in H_2_O. Elution was achieved by a linear gradient of solvent B from 5–50% in 30 min, at a flow rate of 1 mL/min. Quantitative values were obtained by peak integration after UV detection at 280 nm.

## 4. Conclusions

In this study, we present for the first time the biochemical characterization of a recombinantly expressed β-galactosidase from the Pacific oyster, *Crassostrea gigas,* and its comparison with our previously characterized enzyme from the land snail, *Arion vulgaris*. Thereby, the mussel enzyme showed similar biochemical parameters to the snail enzyme. Both enzymes cleaved β1,3 as well as β1,4 linkages with a preference for β1,3. Moreover, the enzymes were inactive in the presence of fucose on the adjacent sugar moiety (3-fucosyllactose and lacto-N-fucopentaose). Using a portfolio of methods (MALDI-TOF MS, HPTLC, HPLC) to determine substrate specificity for both enzymes, we were able to demonstrate the effect of substrate labeling on the enzyme’s activity. We found that the type of label linked to a substrate has a noteworthy impact on its suitability as an acceptor. Hence, fast and sensitive detection methods that do not require substrate labeling to measure glycosidase activity are necessary. While MALDI-TOF MS analysis is a good choice for larger substrates, HPTLC offers a sensitive, affordable, and, above all, a rapid alternative to measure β-galactosidase activity towards small unlabeled molecules.

This study has contributed to characterizing a further member of the huge family of β-galactosidases and has extended our knowledge of the glycosylation machinery in mollusks. Continuing to study the large phylum of mollusks will for sure reveal some biological surprises in the future.

## Figures and Tables

**Table 1:** β-galactosidase activity on unlabeled (HPTLC), AA- or pNP-labeled substrates (HPLC). All results refer to the β-galactosidases from *C. gigas* (NCBI Ref. Nr.: OR180112) and *A. vulgaris* (UniProt Ref. Nr.: A0A0B7AQJ9) [19], as both mollusk enzymes showed identical substrate specificity. ND = not determined.

	HPTLC Unlabeled Substrate	HPLC–AA AA Labeled Substrate	HPLC–pNP pNP Labeled Substrate
Galβ1,4Glc (Lactose)	Yes (partly)	Yes	Yes
Galβ1,3GalNAc (Galacto-N-biose)	Yes	No	Yes
Galβ1,4GlcNAc (N-Acetyllactosamine)	Yes (partly)	No	ND
Galβ1,6GlcNAc	No	No	ND
Fucα1,3[Galβ1,4]Glc (3-Fucosyllactose)	No	No	ND

## Data Availability

Protein sequences are stored at NCBI database.

## Supplementary Materials

The following supporting information can be downloaded at: https://www.mdpi.com/article/10.3390/ijms242015287/s1.
