# Supplementary material for: Expression and Characterization of a β-Galactosidase from the Pacific Oyster, Crassostrea gigas, and Evaluation of Strategies for Testing Substrate Specificity
Source: Int J Mol Sci. 2023 Oct 18;24(20):15287. doi: 10.3390/ijms242015287 (PMC10607238; doi:10.3390/ijms242015287)
Supplement: Supplementary file 1 [file ijms-24-15287-s001.zip › ijms-2652720-supplementary.pdf]

International Journal of
Molecular Sciences

MDPI

Supplementary data

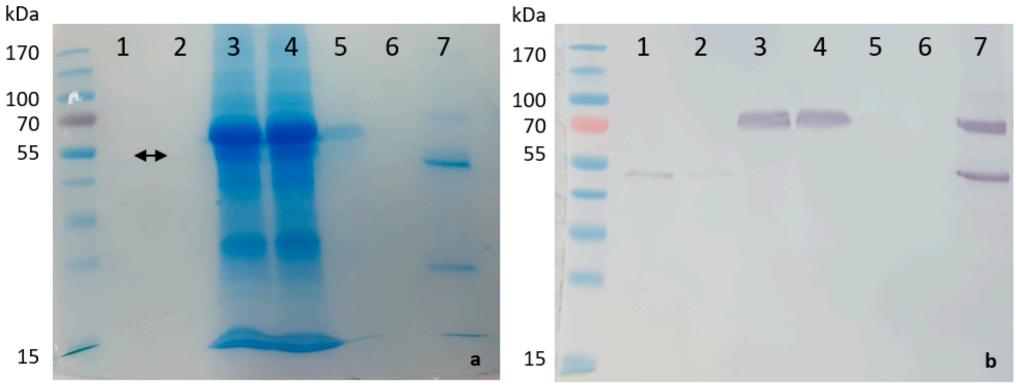

Figure S1. Purification procedure of β-galactosidase from *C. gigas* via immunoprecipitation. (a) SDS-PAGE Coomassie staining, (b) Western Blot using mouse anti-Penta-Histidine tag:HRP monoclonal antibody (1:2500, BIORAD – Vienna, Austria) followed by alkaline phosphatase conjugated anti-mouse IgG from goat (1:4000, Sigma-Aldrich, Vienna, Austria). **(1)** His-tag antibody prior to linkage to agarose beads, **(2)** remaining His-tag antibody in solution after linkage to agarose beads, **(3)** Sf9 lysate fraction containing recombinant β-galactosidase, **(4)** unbound proteins after incubation with agarose beads, **(5)** washing step 1, **(6)** washing step 2, **(7)** purified β-galactosidase (~78 kDa) and heavy chain of mouse anti-Penta-His-tag antibody (~55 kDa).

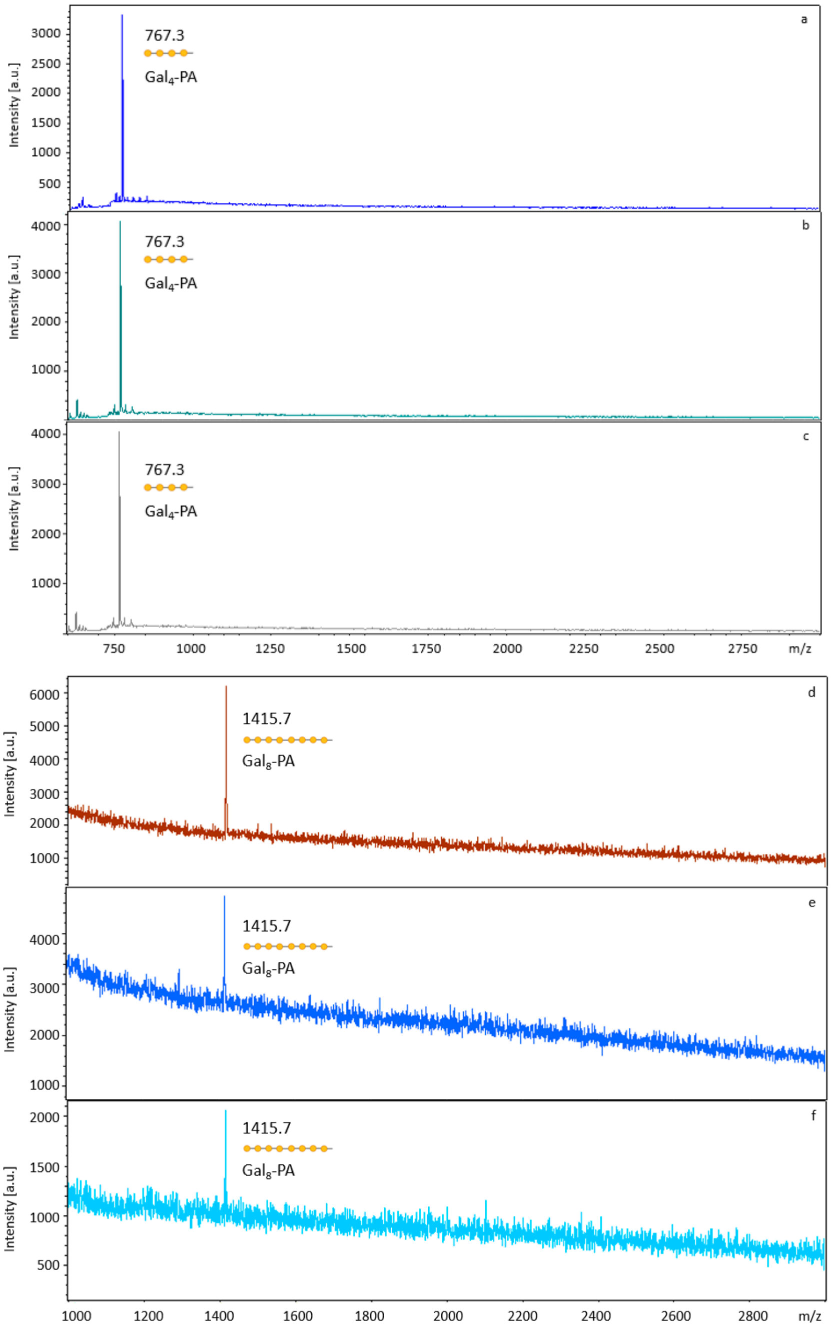

Figure S2 MALDI-TOF analysis of β-galactosidase activity towards galactan chains. (a) Gal4-PA substrate, (b) Gal4-PA incubated with purified β-galactosidase from *C. gigas*, (c) Gal4-PA incubated with purified β-galactosidase from *A. vulgaris*, (d) Gal8-PA substrate, (e) Gal8-PA incubated with purified β-galactosidase from *C. gigas*, (f) Gal8-PA incubated with purified β-galactosidase from *A. vulgaris*. N-Glycan structures were created using bioRENDER

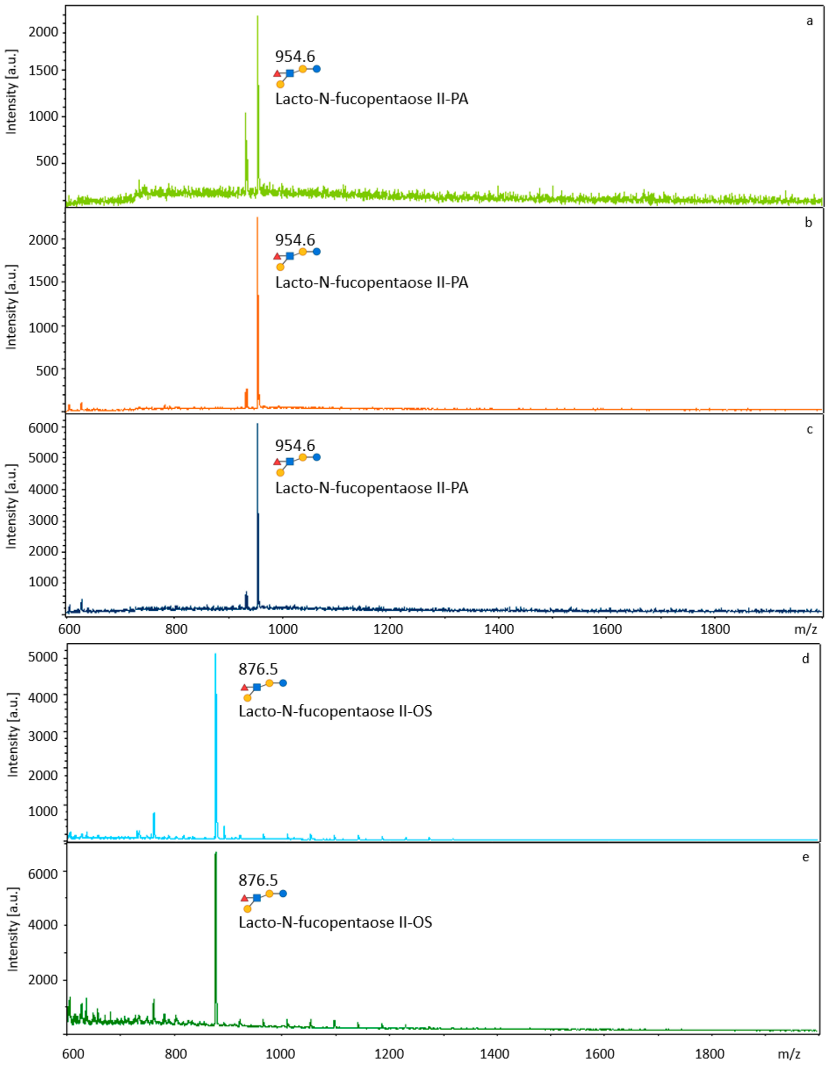

Figure S3 MALDI-TOF analysis of β-galactosidase activity towards lacto-N-fucopentaose II. (a) Lacto-N-fucopentaose II-PA substrate, (b) lacto-N-fucopentaose II-PA incubated with purified β-galactosidase from *C. gigas*, (c) lacto-N-fucopentaose II-PA incubated with purified β-galactosidase from *A. vulgaris*, (d) lacto-N-fucopentaose II-OS substrate, (e) lacto-N-fucopentaose II-OS incubated with purified β-galactosidase from *C. gigas*. N-Glycan structures were created using bioRENDER

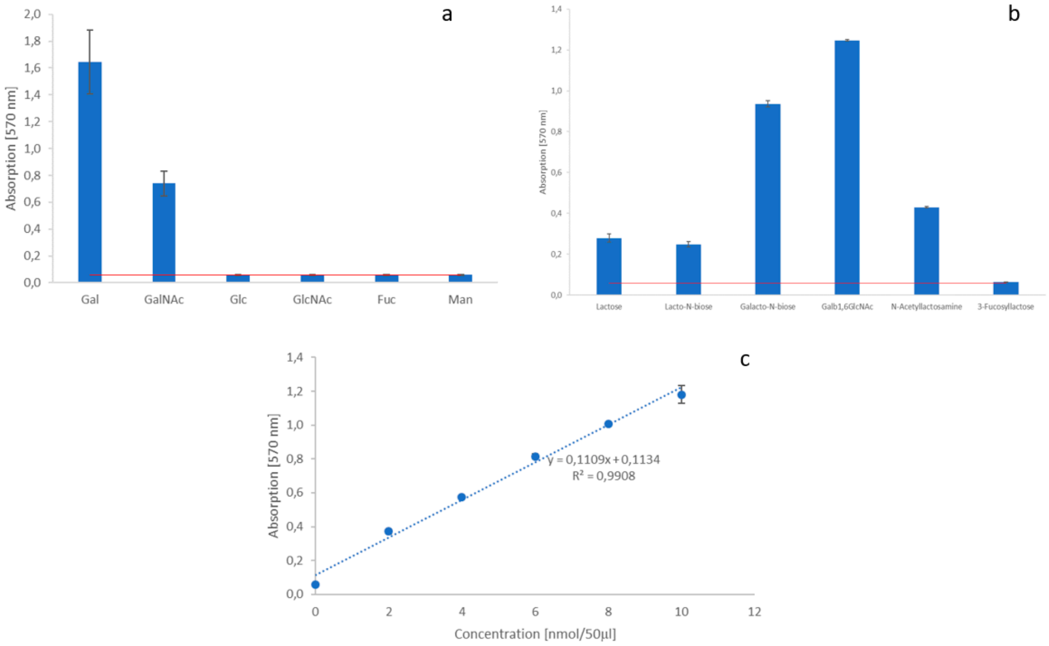

Figure S4. Background of the Galactose Assay Kit (ab83382; ABCAM – Cambridge, UK) with (a) different monosaccharides and (b) di- and trisaccharides without addition of β-galactosidase. Red line depicts a H_2O control without any addition of sugars. (c) Represents the galactose standard calculation curve according to supplier's instructions.

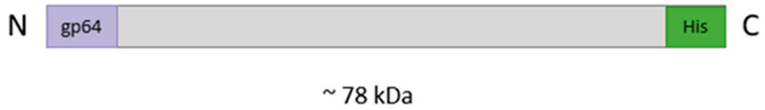

Figure S5. Schematic structure of recombinant full-length β-galactosidase from *C. gigas* (NCBI Ref. Nr.: XP_034310761.1). The recombinant protein consists of gene sequence (grey), N-terminal gp64 leader sequence (purple) for secretion out of Sf9 insect cells and a 6xHis-tag (green) for purification at the C-terminus. Short 3 amino-acid linkers were added to the gene at the N- and C-terminus to connect gp64 leader sequence and 6xHis-tag (not shown).
